# Silver Nanoparticles Modulate the Epithelial-to-Mesenchymal Transition in Estrogen-Dependent Breast Cancer Cells In Vitro

**DOI:** 10.3390/ijms22179203

**Published:** 2021-08-25

**Authors:** Michał Rakowski, Szymon Porębski, Agnieszka Grzelak

**Affiliations:** 1 The Bio-Med-Chem Doctoral School of the University of Lodz and Lodz Institutes of the Polish Academy of Sciences, University of Lodz, 90-237 Lodz, Poland; 2 Cytometry Laboratory, Department of Molecular Biophysics, Faculty of Biology and Environmental Protection, University of Lodz, 90-236 Lodz, Poland; szymon.porebski@edu.uni.lodz.pl

**Keywords:** silver nanoparticles, breast cancer, epithelial–mesenchymal transition, metastasis, estrogen, MCF-7

## Abstract

Silver nanoparticles (AgNPs) are frequently detected in many convenience goods, such as cosmetics, that are applied directly to the skin. AgNPs accumulated in cells can modulate a wide range of molecular pathways, causing direct changes in cells. The aim of this study is to assess the capability of AgNPs to modulate the metastasis of breast cancer cells through the induction of epithelial-to-mesenchymal transition (EMT). The effect of the AgNPs on MCF-7 cells was investigated via the sulforhodamine B method, the wound healing test, generation of reactive oxygen species (ROS), the standard cytofluorimetric method of measuring the cell cycle, and the expression of EMT marker proteins and the MTA3 protein via Western blot. To fulfill the results, calcium flux and HDAC activity were measured. Additionally, mitochondrial membrane potential was measured to assess the direct impact of AgNPs on mitochondria. The results indicated that the MCF-7 cells are resistant to the cytotoxic effect of AgNPs and have higher mobility than the control cells. Treatment with AgNPs induced a generation of ROS; however, it did not affect the cell cycle but modulated the expression of EMT marker proteins and the MTA3 protein. Mitochondrial membrane potential and calcium flux were not altered; however, the AgNPs did modulate the total HDAC activity. The presented data support our hypothesis that AgNPs modulate the metastasis of MCF-7 cells through the EMT pathway. These results suggest that AgNPs, by inducing reactive oxygen species generation, alter the metabolism of breast cancer cells and trigger several pathways related to metastasis.

## 1. Introduction

The uncontrolled spread of cancer cells in the body (metastasis) often results in rigorous therapy and frequently leads to the death of the patient. The risk factors for developing cancer are environmental factors (such as an unhealthy diet or lack of physical activity), exposure to pollutants, and possession of genetic predispositions.

Breast cancer, the incidence of which is often influenced by age [1], has rapidly become the most commonly diagnosed cancer in women [2]. Primary factors that induce the development of breast cancer are generally associated with increased exposure to estrogens, early adolescence, late menopause, late age of first pregnancy, forsaking breastfeeding [3], and taking contraceptives [4]. Most malignant tumors found in breasts contain estrogen receptors (ERs) and progesterone receptors (PRs). In terms of ER expression, breast cancer tumors can be classified into two types: ER-positive (ER+) and ER-negative (ER-). It has been reported that younger women more frequently have ER- cancer [5]. In addition to age, the decisive factors for developing estrogen-dependent breast cancer are menopause, obesity, use of contraceptives, and hormone replacement therapy (HRT) during menopause [6]. The distinguishing feature of ERs is low substrate specificity, resulting in ER binding not only endogenous estrogens but also structurally distant synthetic compounds as well as secondary metabolites of higher plants.

The binding of exogenous ligands does not always lead to the inducement of estrogen signaling. It can cause a disruption of estrogen pathways, block the ligand-binding site, or activate a different signaling pathway. These changes may lead to many serious health problems, such as the aforementioned metastasis. Epithelial-to-mesenchymal transition (EMT) and its reverse process, mesenchymal-to-epithelial transition (MET), are key for the development of many tissues and organs [7]. EMT is a biological process that allows a polarized epithelial cell with a normal epithelial phenotype to undergo multiple biochemical changes, enabling it to adopt a mesenchymal phenotype associated with increased cell migration capacity, increased resistance to apoptosis, and significantly increased production of extracellular matrix (ECM) components [8]. Epithelial cells are characterized by high levels of E-cadherin, while cells with a mesenchymal phenotype are rich in N-cadherin, fibronectin, and vimentin. Thus, EMT involves deep morphological and phenotypic changes in the cell. As a result of this process, the basement membrane is degraded, and the cell phenotype shifts to the mesenchymal type, enabling migration from the epithelial layer from which it originates. It is believed that the basic indicator that occurs during EMT is lower than the physiological level of E-cadherin. Proteins such as Snail 1, Snail 2 (often called Slug), ZEB1, ZEB2, TCF3, and KLF8 can attach to the E-cadherin promoter and inhibit its transcription [9].

Due to the low substrate specificity, the ERs can bind a lot of extracellular components, often called estrogen mimetics. The estrogen mimetics can be divided into two separate groups based on their origin: phytoestrogens and metalloestrogens. The first group comprises a plethora of natural compounds produced by plants, whereas the second’s structure is based on elemental metal. The group of metalloestrogens includes all forms of elements from the metal group, ranging from ions to nanoparticles.

Silver nanoparticles (AgNPs) are the most commonly used nanoparticles in household products (as an alternative to detergents and fungicides) due to their bactericidal and fungicidal properties. The introduction of nanomaterials into the environment in recent decades has resulted in continuous accumulation of the nanomaterials in the abiotic environment and, consequently, in the tissues of organisms. Only in the last decade have discussions begun concerning the long-term effects of mass and continuous exposure of organisms to nanomaterials and how great a burden this is for the abiotic environment [10].

Nanoparticles are known for their interactions with molecular pathways in the cell. There are dozens of published papers about green-synthesized AgNPs that are modified or unmodified with organic or inorganic compounds and can modulate many various pathways in breast cancer cells. Depending on the modification of the surface, the AgNPs present a different mechanism of action. Despite the positive sides of the AgNPs, these nanoparticles can also cause (depending on their size, surface modifications, shape) cytotoxicity, mitochondrial dysfunction, and oxidative stress and induce apoptosis [11] and—as shown in this paper—metastasis through induction of the EMT pathway. The exact background of our hypothesis that AgNPs cause metastasis in breast cancer cells was our own observation of MCF-7 cells and the already proven low specificity of estrogen receptors that can bind a plethora of ligands [12].

In this paper, we wanted to present the results of experiments conducted to verify the hypothesis about the metastasis caused by AgNPs in estrogen-dependent breast cancer cells of the MCF-7 cell line, which is the most commonly used and well-described in vitro model of ER+ breast cancer. We believe that our research will contribute to an improved understanding of the complex network of connections between nanomaterials and intracellular transmission pathways, with particular emphasis on implications for the treatment of hormone-dependent breast cancers.

## 2. Results

All experiments were conducted on MCF-7 cells cultured in Dulbecco’s modified Eagle medium (DMEM) supplemented with 10% fetal bovine serum (FBS). All experiments were conducted at least in triplicate (*n* = 3). The statistics were analyzed (unless otherwise stated) via Student’s *t*-test by comparing control cells with treated cells (α = 0.05) via GraphPad Prism 8 software. Statistical significance was formatted as follows: * *p* ≤ 0.05, ** *p* ≤ 0.01, *** *p* ≤ 0.001.

### 2.1. Survival of the MCF-7 Cells Treated with AgNPs

To measure the cytotoxicity of AgNPs, the fluorimetric assay based on sulforhodamine B (SRB), a dye that binds to cellular proteins, was used. This method is insensitive to the turbidity of the sample, which could occur due to the release of AgNPs accumulated in cells. Commonly used methods for measuring the viability of cells, such as MTT, could provide a false-positive result due to the content of AgNPs in the sample. Thus, the SRB assay provides reliable viability results.

**Figure 1:**
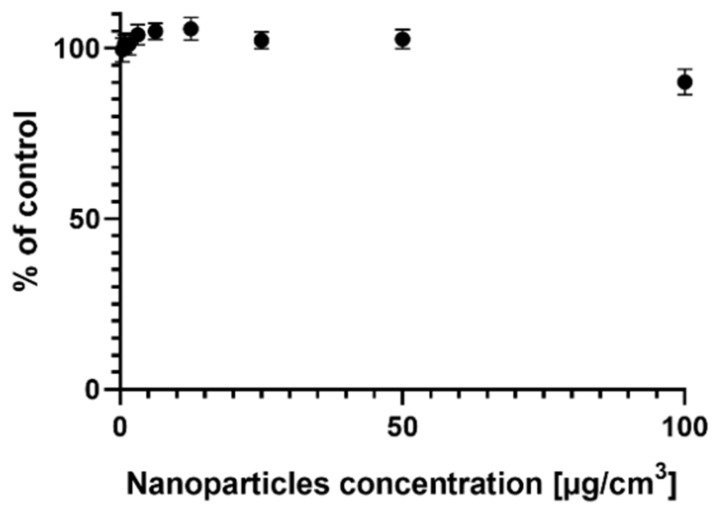
Viability of the MCF-7 cells treated with silver nanoparticles (AgNPs) at a range of concentrations for 24 h.

Figure 1 shows the results of the survival of MCF-7 cells treated with AgNPs at a range of concentrations. The results are presented in comparison with the control cells. Silver nanoparticles in the range of 0.39–100 µg/cm^3^ did not cause acute toxicity in MCF-7 cells after 24 h of incubation. It was not possible to determine the IC_50_ concentration of AgNPs because even at the highest used concentration (100 µg/cm^3^), the viability of the cells was much higher than 50%.

### 2.2. Assessment of the Migration Capacity of MCF-7 Cells via Wound Healing Test

The EMT is closely related to a change in the migration of cells. Degradation of the basal membrane and a change in the protein profile of the cells will lead to a shift in their proliferation and migration capacity. In the commonly used wound healing test, the cell monolayer is damaged, and the percent of the wound filled by cells after incubation is measured.

**Figure 2:**
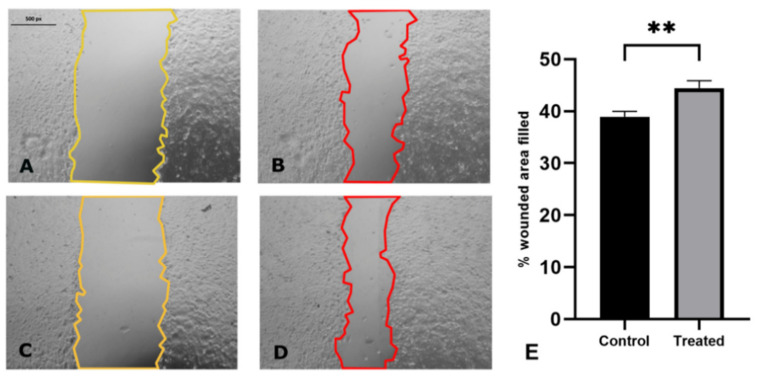
Images showing the wound healing test conducted on MCF-7 cells. (**A**) Control cells at time 0; (**B**) control cells after a 24 h incubation from scratching; (**C**) cells before adding AgNPs at time 0; (**D**) cells after a 24-h incubation with AgNPs (25 µg/cm^3^). Pictures (**A**,**B**) and (**C**,**D**) were taken in the same appropriately marked location of each scratch in the cell monolayer. The scratch surface was analyzed with a Nikon Eclipse Ti microscope, and the scratch surface area was expressed in pixels (px). Graph (**E**) presents the results of the wound healing assay for MCF-7 cells treated with AgNPs at a concentration of 25 µg/cm^3^ for 24 h.

Figure 2 shows the photographs of cell cultures before (on the left, marked in yellow) and 24 h after (on the right, marked in red) wounding the cell monolayer. A scratch was made in the cell culture monolayer after it reached 100% confluence. The samples were rinsed thoroughly to remove the detached cells, and photographs of the culture were taken under a light microscope after the addition of AgNPs. Photographs were taken again, in the same location, after the 24-h incubation period. The results are presented as the ratio of the scratch area before and after the incubation of the cells, expressed as a percentage of the area filled. Cells treated with AgNPs (Figure 2C,D) displayed improved results in the scratch test when compared to the control cells (Figure 2A,B). Cells after 24 h of incubation with AgNPs (25 µg/cm^3^) showed a greater ability to migrate (44.46% of the wounded area filled) when compared to the control cells (38.95% of the wounded area filled), which was statistically significant (*p* < 0.01). The MCF-7 cells treated with AgNPs are characterized by an improved ability to overgrow the scratch in the cell monolayer.

### 2.3. Reactive Oxygen Species (ROS) Generation

**Figure 3:**
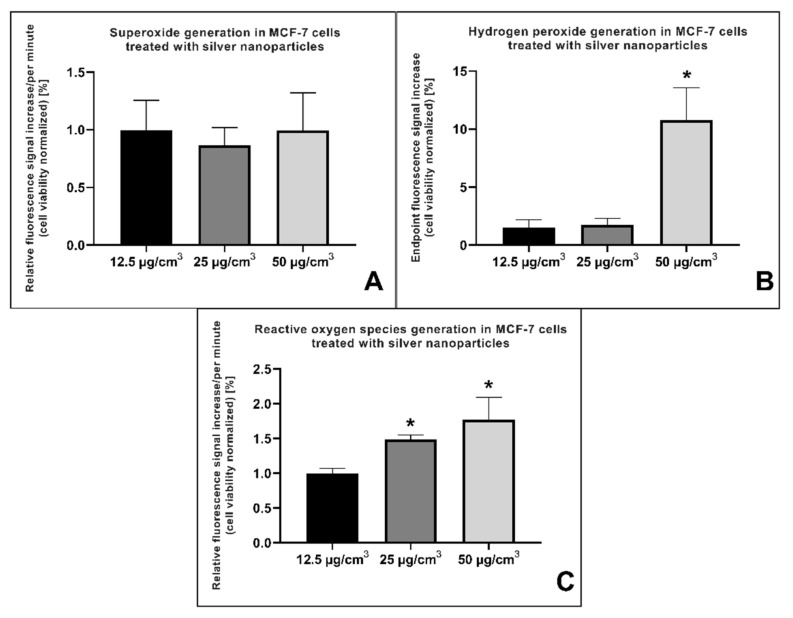
Reactive oxygen species generation in MCF-7 cells after 24 h treatment with silver nanoparticles in three different concentrations—50, 25, and 12.5 µg/cm^3^. (**A**) The generation of superoxide measured with a dihydroethidium (DHE) fluorescent probe. The amount of generated hydrogen peroxide (**B**) was measured with a dihydrorhodamine 123 (DHR123) fluorescent probe. Finally, the overall amount of ROS (**C**) was measured with a 2′,7′-dichlorodihydrofluorescein diacetate (H_2_DCFDA) fluorescent probe. The presented data were normalized to cell viability and compared with the control.

Silver nanoparticles are known for their ability to induce reactive oxygen species (ROS) generation in mammalian cells. The amount of generated ROS highly depends on the type of the cell, its metabolic activity (i.e., amount of mitochondria), the redox status of the environment, and many other variables that influence the AgNP-induced ROS generation. Below, the data from a series of experiments using three different fluorescent probes—DHR123, DHE, and H_2_DCFDA—is shown (Figure 3).

As shown in Figure 3A, the amount of generated superoxide in MCF-7 cells after 24 h treatment with AgNPs in the range of concentrations did not differ significantly compared to the control. The amount of generated hydrogen peroxide—which is a product of superoxide dismutation—was statistically significant (*p* < 0.05) after 24 h treatment with AgNPs in a concentration of 50 µg/cm^3^ (Figure 3B). The overall amount of generated ROS after treatment with AgNPs is shown in Figure 3C and was measured via H_2_DFCDA-induced fluorescence signal. The data clearly show that AgNPs in the concentration of 25 and 50 µg/cm^3^ after 24 h of incubation time induced a generation of ROS that was statistically significant (*p* < 0.05).

### 2.4. Cell Cycle Measurement

The EMT, in addition to changes in cell migration, can also be associated with a change in the proportion of cell cycle phases. We examined the effect of AgNPs on the cell cycle of MCF-7 cells. MCF-7 cells were cultured in 6-well plates for 24 h. AgNPs were then added to the cells at a final concentration of 25 µg/cm^3^ for an additional 24 h. The cells were then subsequently prepared for cytofluorimetry measurement (as described in the Section 4).

**Figure 4:**
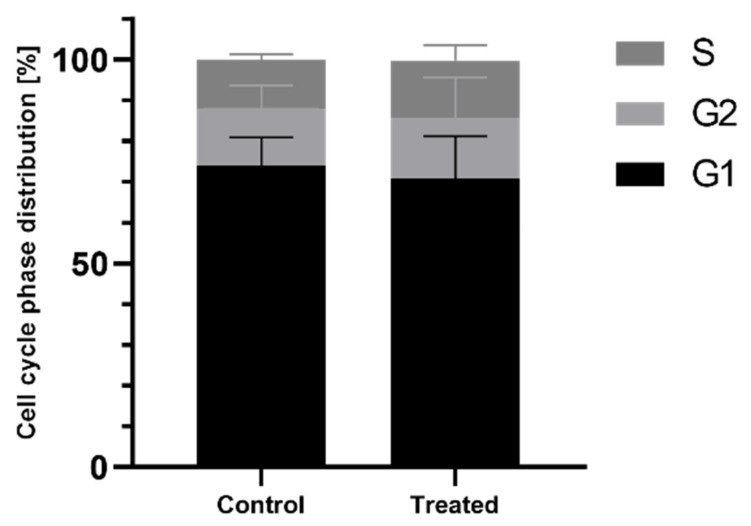
Cell cycle phase distribution of MCF-7 cells treated with AgNPs at a concentration of 25 µg/cm^3^ for 24 h.

The results are summarized in Figure 4. The data presented in the graph is expressed as a percentage of the number of cells present in each phase of the cell cycle. In the control culture, there were more cells in the G1 phase when compared to the cells incubated with AgNPs. The number of cells in the G2 and S phases was greater in the culture incubated with AgNPs when compared to the control cells. However, the differences were not statistically significant. Thus, it can be concluded that AgNPs, at a concentration of 25 µg/cm^3^, do not modulate the cell cycle in MCF-7 cells.

### 2.5. Measurement of EMT Marker Proteins and MTA Protein Levels

During EMT, tremendous changes occur in the protein profile of the cells. This is necessary for the cells to alter their morphology from epithelial to mesenchymal and detach from the basal membrane. Several proteins are recognized as EMT markers: Slug, Snail, vimentin, and N-cadherin are the most described. In the present study, we analyzed a range of EMT markers: vimentin, N-cadherin, Claudin-1, β-catenin, ZO-1, Snail, Slug, ZEB1, and E-cadherin. The loss of E-cadherin is thought to be the primary marker of the EMT process. The MCF-7 cells used in this study did not express all of the above-mentioned proteins; our culture showed expression of E-cadherin, β-catenin, Snail, and ZO-1.

**Figure 5:**
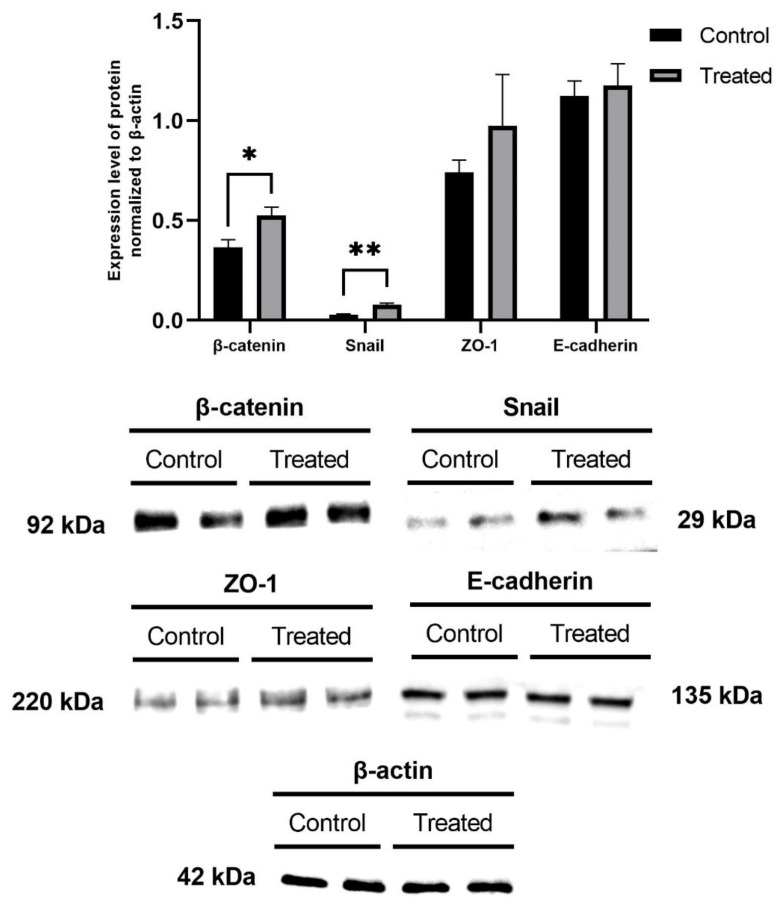
Expression of EMT marker proteins in MCF-7 cells treated with AgNPs at a concentration of 25 µg/cm^3^ for 24 h. The chemiluminescence photos of protein bands were derived from one gel/membrane and were physically cut before the visualization due to huge differences in the protein levels.

The expression level of EMT marker proteins in MCF-7 cells was analyzed via Western blot. After 24 h of incubation with AgNPs at a concentration of 25 µg/cm^3^, MCF-7 cells were lysed with M-PER solution. Figure 5 shows the expression of EMT marker proteins in MCF-7 cells after incubation with AgNPs. The cells treated with AgNPs showed a higher level of expression of the β-catenin protein when compared to the control cells (*p* < 0.05), which was similar in the case of the Snail protein (*p* < 0.01). There were no significant changes in the level of E-cadherin and ZO-1 proteins.

**Figure 6:**
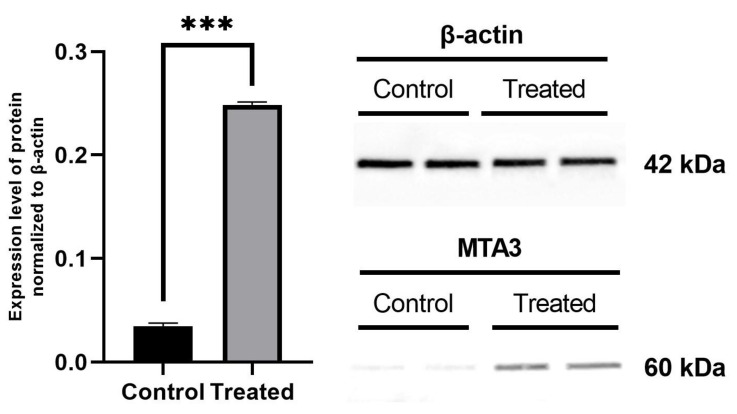
Expression of MTA3 protein in MCF-7 cells treated with AgNPs at a concentration of 25 µg/cm^3^ for 24 h. The chemiluminescence photos of protein bands are derived from one gel/membrane and were digitally cut using an image editor to show the differences between protein levels.

The MTA3 protein level in MCF-7 cell lysates was significantly higher for AgNP-treated cells compared with control (Figure 6; *p* < 0.001). When normalized to β-actin, the results revealed that the cells treated with AgNPs had an approximately 6-fold increase in the MTA3 protein level compared to control cells.

### 2.6. Assessment of Mitochondrial Membrane Potential

It is well-proven that AgNPs’ mechanism of action highly depends on the generation of ROS. Mitochondria of the mammalian cells are especially susceptible to the direct damage induced by AgNPs; thus, we decided to measure the membrane potential of mitochondria in MCF-7 cells after treatment with AgNPs in a range of concentrations.

**Figure 7:**
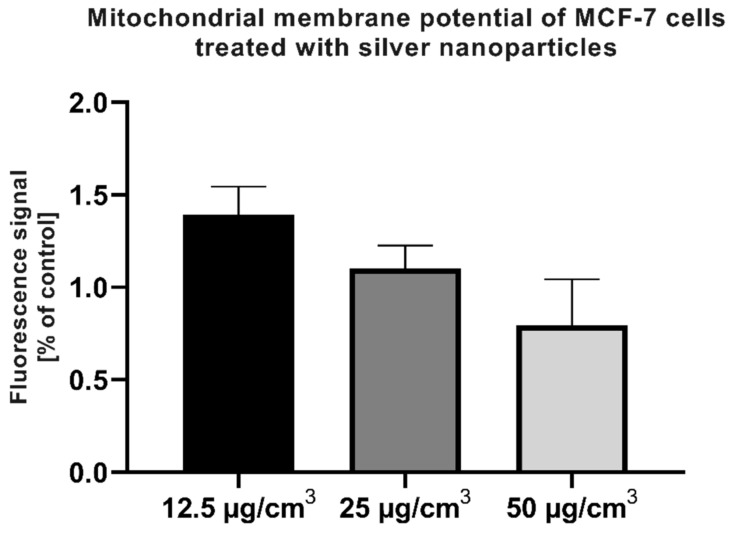
Mitochondrial membrane potential in MCF-7 cells after 24-h treatment with AgNPs in a range of concentrations, measured with a Mitotracker™ Red CMXRos probe.

As shown in Figure 7, the mitochondrial membrane potential of MCF-7 cells treated with AgNPs did not differ significantly compared to the control cells.

### 2.7. Measurement of the Intracellular Calcium Level after the Addition of AgNPs

Fura-2 is a fluorescent Ca^2+^ indicator often used to measure cytoplasmic calcium levels. The fluorescence of Fura-2 in its original state can be measured with 380 nm excitation and 510 nm emission wavelengths, whereas Fura-2 bound with Ca^2+^ shifts its excitation spectrum toward a shorter wavelength, i.e., 340 nm. Therefore, the 340/380 nm fluorescence signal ratio indicates the change in the intracellular level of Ca^2+^.

**Figure 8:**
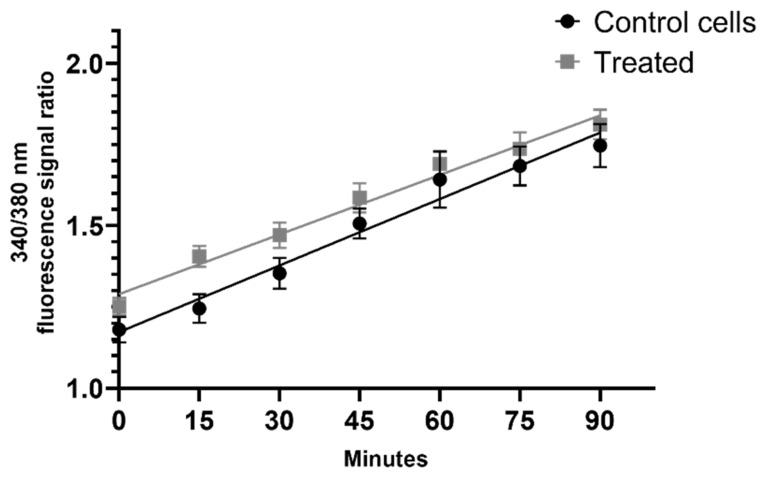
Calcium flux after the addition of silver nanoparticles at a concentration of 25 µg/cm^3^. The graph presents the ratio of Fura-2 fluorescence intensity at 340 and 380 nm wavelengths. Cells were measured immediately after the addition of AgNPs for 120 min (the measurement was done every 15 min).

Our experiments showed no difference in intracellular calcium level between the control and treated cells (Figure 8). The 95% confidence intervals for slopes were 0.005235 to 0.007524 and 0.005155 to 0.006659 for control and treated cells, respectively. Best-fit values, calculated by GraphPad Prism 8, were 0.006379 for control and 0.005907 for treated cells. Analysis of regression showed no statistical significance between the slopes (F = 0.4676; *p* = 0.4948).

### 2.8. Histone Deacetylase (HDAC) Activity

Histone deacetylases modulate vast molecular processes through their repressive influence on transcription. Alteration in the activity of HDAC often significantly impacts cellular signaling related to metastasis.

**Figure 9:**
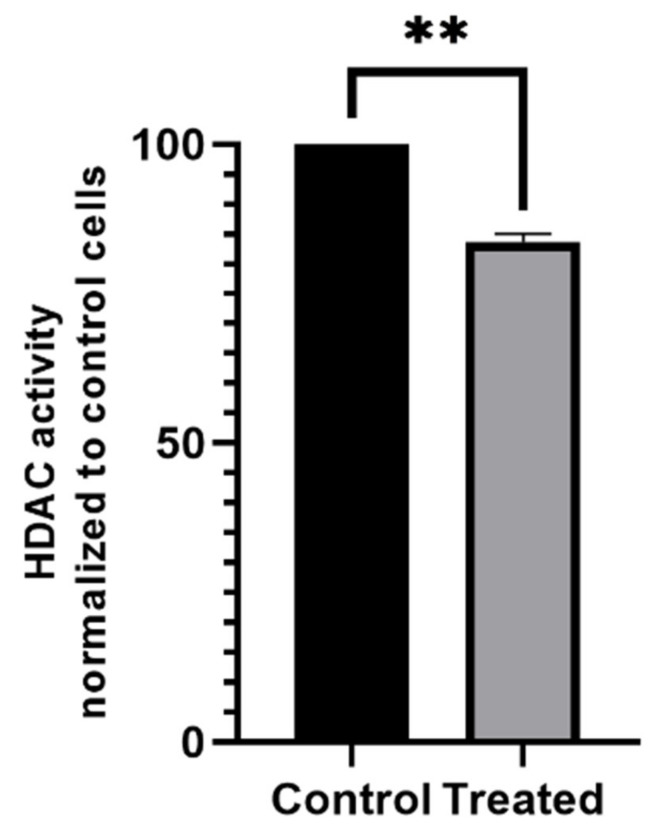
Activity of HDAC after treatment with AgNPs. The graph presents the activity of HDAC from MCF-7 cell nuclei crude extract that was incubated with AgNPs at a concentration of 25 µg/cm^3^.

As shown in Figure 9, the MCF-7 cells after treatment with AgNPs had lower activity of HDAC when normalized and compared to control cells. Thus, AgNPs at a concentration of 25 µg/cm^3^ after 24 h of incubation did change the HDAC activity in MCF-7 cells in an E2-deprivation setup.

## 3. Discussion

Silver nanoparticles are widely used in medicine and pharmacology. They enter the human environment intentionally as an additive in cosmetics, antibacterial agents, or bandages. Because of their unique physicochemical properties and well-understood mechanism of toxicity, the AgNPs are often used in therapeutic approaches, most often as drug carriers. Several studies have described the effect of green-synthesized AgNPs on MCF-7 cells. The MCF-7 cell line is a well-established and willingly used hormone-dependent human breast cancer cell model used in the basic research because of the physiological expression of estrogen receptor α. This particular cell line is—among other applications—often used in research on E2-dependent cellular signaling pathways and interactions between drugs and potential estrogen mimetics. The estrogen mimetics can be plant-derived metabolites, environmental pollutants (such as cadmium [13]), or elemental metals. Nanoparticles are a member of the metal estrogen mimetics family; thus, the mechanism of action of the AgNPs is different in hormone-dependent cells than in triple-negative cell lines [14]. These differences are most probably caused by the low specificity of the estrogen receptor that can bind a vast number of ligands, each causing a different effect, i.e., promoting cellular signaling, blocking the receptor binding site, and so forth. In the aforementioned papers, the results suggest that the AgNPs exert an action similar to estrogen mimetics through the activation of ERα-dependent cellular signaling pathways. Furthermore, we assumed that the ROS-generating ability of AgNPs would influence the morphology of the cells cultured in the E2-deprivation setup, thus leading to their detachment and an increase in migration potential. The experimental conditions utilized in the present study simulate the conditions of estrogen deprivation, under which breast cancer cells are found in the body of postmenopausal women.

Our research, from the very beginning, was meant to check if the AgNPs would induce an effect similar to that induced by estrogen mimetics. A great majority of published papers is based on hormone-dependent cell lines such as MCF-7, cultured without the addition of E2 in media supplemented with phenol red, which, in fact, also is an estrogen mimetic [15]. Thus, the deprivation of E2 in that particular situation is being compensated by the addition of phenol red. In vivo, the cells are constantly exposed to many endogenous and exogenous substances, of which some of them can be estrogen mimetics.

Silver nanoparticles are commonly described as cytotoxic in vitro. Their toxicity depends on the cell line used and the size of the nanoparticles [16]. Discussions on AgNPs can be problematic because literature reports are very diverse. Most of the published papers are based on green-synthesized AgNPs [17], which often contain a relevant amount of silver ions [18]. In the case of clean, properly dispersed AgNPs, coated with protein coronas, the problem with silver ion residue is negligible because the amount of released intracellular silver ions is minimal. Cronholm et al. [19] showed that silver and copper nanoparticles are more toxic than the salts of these elements. The authors suggest that the mechanism differentiating these two forms means that nanoparticles are being absorbed by the cells. They verified this hypothesis using laser scanning confocal microscopy (LSCM) and transmission electron microscopy (TEM) methods and confirmed that after only 4 h of incubation, the nanoparticles were delocalized into the intracellular matrix. The exact localization of silver and copper nanoparticles in the cells was the same, but after another 20 h of incubation, there were only AgNP aggregates inside the cells. The IC_50_ parameter for AgNPs is also problematic because it differs significantly between published papers and usually ranges from several to even a 100 μg/cm^3^. In addition, most papers do not provide information on the confluence of the cell monolayer, which is an important parameter because the ratio of the amount of AgNPs to cell area affects the endpoint of the experiment.

Mammalian cells in vitro release many substances into the extracellular matrix: proteins, metabolites, and compounds that affect the pH of cell media. Our aim is to assess the impact of AgNPs on those substances released to the conditioned medium, which would change the redox status of the medium; therefore, the use of methods based on the reduction of resazurin or MTT [17,20] is not a reliable method to measure cell survival. Redox status of the extracellular environment is dynamic, shaped by intracellular metabolism. A lot of signaling pathways are involved in this process, especially autocrine and paracrine signaling, which directly affect intercellular signaling. For example, mammalian cells actively secrete sulfhydryl oxidases that catalyze the reaction of reducing molecular oxygen to hydrogen peroxide, which is involved in cell signaling, regulation of cell adhesion, and proliferation [21].

Resazurin is commonly used as an indicator of cell viability. It penetrates the membrane and is enzymatically reduced in the presence of microsomes to resorufin. During the reduction process, the electrons are transferred from NADPH+H^+^ to resazurin. Research conducted on *Enterococcus faecalis* showed that the reduction of resazurin occurs only inside the cells; when the cells were sonicated and, thus, the enzymes and metabolites were released, the reduction of resazurin still occurred, even without live cells [22]. In turn, a paper by O’Brien et al. [23] clearly shows that in mammalian cultures, resazurin is reduced inside the cells or through the reaction of resazurin with conditioned media.

The MTT test is based on the conversion of MTT to formazan crystals by the mitochondria of live cells, determining their activity. Because in most cell cultures, the activity of mitochondria is relative to the viability of the cells, this test is widely used to assess the cytotoxic influence of drugs on the cell lines in vitro. A paper by Bernas and Dobrucki [24] shows that MTT is actively absorbed by the cells and easily undergoes the reduction catalyzed by membrane reductases and intracellular enzymes. The authors suggest that only a part of the crystalized formazan is localized on the surface of the cell and inside the mitochondria when the same reaction occurs simultaneously in other parts of the cells such as the cytoplasm or cell membrane. The results of our study demonstrate that commercially available AgNPs, with a diameter of ~20 nm, do not exert acute cytotoxicity on MCF-7 cells (Figure 1). To assess the viability of the MCF-7 cells, we used a quantitative method based on the binding of sulforhodamine B to cell proteins. It is a fluorometric method; thus, measurement interferences due to the turbidity of the sample are excluded (as in the case of methods based on the absorbance of the sample), and it is not burdened with uncertainty, as in the case of methods based on the reduction of substrates.

The initial symptom of EMT that can be observed (using a microscope) is the migration capacity of cells, which is manifested, inter alia, in the popular and widely used scratch overgrowth test. Rodríguez-Razón et al. [25] studied the migration ability of breast cancer cell lines and compared the effect of AgNPs (of 2–9 nm nominal diameter) on three breast cancer cell lines: MCF-7, HCC70, and HCC1954. The AgNPs significantly reduced the adhesion of MCF-7 cells and decreased the viability of the cells by approximately 45%. The scratch test, commonly used to assess the migration capacity of the cell culture, is based on creating a wound in a monolayer of cells and its observation over time. For example, green-synthesized AgNPs, with a nominal diameter of ~48 nm, have been shown to inhibit the migration of MCF-7 cells dose-dependently [26], while our results demonstrate that AgNPs with a nominal diameter of 20 nm promote cell migration at a concentration of 25 µg/cm^3^ (Figure 2). It is widely acknowledged that the cytotoxicity and overall biological effect induced by AgNPs is size-dependent; however, a single principle underlies this: smaller nanoparticles are generally more toxic than larger nanoparticles due to a greater ability to penetrate cells [27,28,29]. AshaRani et al. [30] reported that AgNPs at a concentration of 25 µg/cm^3^ did not cause cytotoxicity in lung fibroblast cells (IMR-90), and the uptake of the nanoparticles occurred primarily through clathrin-dependent endocytosis. The results indicated that AgNPs induce mitotic arrest in normal human fibroblasts, which corroborates the results obtained in the present study, i.e., treatment with AgNPs did not influence the MCF-7 cells (Figure 4). AshaRani et al. [30] also postulated that the cytotoxicity of AgNPs was due to the formation of intracellular calcium (Ca^2+^) transients in the cells. Li et al. [31] used silver nanoparticle/chitosan oligosaccharide/poly(vinyl alcohol) (PVA/COS-AgNP) nanofibers to check their effect on fibroblasts from human skin. The PVA/COS-AgNP nanofibers upregulated cell factors associated with the TGF-β1/Smad signal transduction pathway, resulting in accelerated wound healing that was inhibited by the TGF-β receptor inhibitor SB431542. The authors suggest that the stimulation of the cells with prepared nanofibers induced the TGF-β1/Smad signal transduction pathway, resulting in the promotion of migration of the cells. Seo et al. [32] observed the effect of AgNPs on wound healing in *Danio rerio* (zebrafish) using the AgNPs of 72.66 nm nominal diameter. The AgNPs caused a visually faster wound healing and the upregulation of wound-healing-related genes, namely, TGF-β, MMP-9 and MMP-13, IL-1β, TNFα, as well as antioxidant enzymes superoxide dismutase and catalase.

The AgNPs’ mechanism of action in most cases is based on the generation of ROS, which triggers intracellular signaling pathways. Khan et al. [17] showed that green-synthesized AgNPs, in a concentration of 5 μg/cm^3^, increase the intracellular production of ROS in MCF-7 cells by 200% when compared to control. This amount of AgNPs was enough to induce a strong apoptotic response, resulting in a 2-fold increase in the expression of Caspase-3. Furthermore, Ullah et al. [33] reported that green-synthesized AgNPs in a concentration of 12.35 μg/cm^3^ increased ROS production dose-dependently and increased the activity of Caspase-3 and Caspase-9. Oxidative stress is an inherent attribute of mammalian cell cultures treated with AgNPs [34], with some exceptions such as pneumocytes, which have a high tolerance to oxidative stress and a well-established antioxidant defense system (data not shown).

In our paper, we have shown the generation of ROS in MCF-7 cells treated with AgNPs in a range of concentrations (Figure 3). To measure the generation of free radicals, a set of fluorescent probes was used: DHR123, DHE, and H_2_DCFDA. The measurement of superoxide (which, in mammalian cells, is a precursor to other ROS) after 24 h incubation with AgNPs did not show a statistical difference between the treated cells and the control (Figure 3A). Additionally, we did not detect the superoxide after a shorter time of incubation (8 and 4 h) with AgNPs (data not shown), most probably because the oxidative stress induced by AgNPs in MCF-7 cells does not—at the beginning—exceed the adaptive level of oxidative stress. Moreover, an efficiently working antioxidant system, consisting mainly of superoxide dismutase, may also be the cause of a lack of statistical significance. The fact that the oxidative stress generated in this cell line is low may be evidenced by the relatively high resistance of the MCF-7 cell line to the AgNPs (Figure 1), which, at the same time, confirms the key role of oxidative stress in AgNP cytotoxicity.

After 24 h of incubation with AgNPs, the MCF-7 cells showed a higher level of total ROS compared to the control (Figure 3C). These data confirm that the adaptive capacity of the cells was exceeded, and, thus, the disruption of redox equilibrium led to the generation of ROS. Similar results were generated with the DHR123 fluorescent probe (Figure 3B); however, the lower concentrations of AgNP (i.e., 12.5 and 25 μg/cm^3^) did not differ significantly from the control cells. These differences are most probably because of the higher specificity of DHR123, which mainly detects H_2_O_2_, while H_2_DCFDA detects a total level of ROS [35]. Undetectable levels of superoxide (Figure 3A) prove that in our setup, the ROS are most probably not being generated directly from mitochondria; however, the total level of ROS is sufficient to activate the intracellular signaling pathways.

A place mainly responsible for the generation of ROS in mammalian cells is the mitochondria. Mitochondrial membrane potential is a key parameter when assessing the damage caused directly to these organelles. In our setup, the AgNPs did not modulate the mitochondrial membrane potential (Figure 7) after 24 h incubation. These results suggest that the mitochondria are not being directly damaged in this particular setup, which corresponds to the lack of difference in calcium flux (Figure 8) after the treatment with AgNPs.

As reported by Gorowiec et al. [36], oxidative stress can induce EMT via a TGF-β1-dependent signaling pathway. The authors showed the upregulation of EMT markers (vimentin, αSMA, fibronectin, and pro-collagen type III) in an in vitro model of alveolar epithelium after incubation with hydrogen peroxide. Furthermore, they reported an increase in the secretion of pro-MMP-2 and active MMP-2 in response to oxidative stress. High MMP-2 expression has been associated with poor prognosis and the induction of EMT in nasopharyngeal carcinoma [37]. During EMT, the cell undergoes genomic processes, including the activation of transcription factors and changes in the expression of miRNAs as well as non-genomic processes involving the release of MMPs, cytoskeletal reconfiguration, and the expression of proteins involved in EMT [38]. Then, a number of transcription factors are activated, which trigger multiple signaling pathways and molecules such as Akt, MAPK, STAT3, TGF-β, β-catenin, Wnt, Ras, Notch, NF-κB, and TNF-α [39,40]. Snail protein has a strong effect in increasing the invasiveness of the cells. It has been previously reported in a hepatocellular carcinoma model in vitro that the MMP gene family is upregulated by Snail expression, which is positively correlated with the upregulation of invasion in HepG2 cells [39,41]. Azhar et al. [42] reported that green-synthesized AgNPs cause oxidative stress in HepG2 cells and lead to cell death through the association with MMP loss apoptosis. Furthermore, Agraval and Yadav [43] showed an increase of MMP-2 and MMP-9 by cigarette smoke extract in A549 cells, which, in turn, triggered the molecular signaling cascade through the EGFR/AKT/ERK/β-catenin axis. Presented changes were restored by the addition of MMP-2 and MMP-9 inhibitors. Our results present the upregulation of Snail and β-catenin in cells treated with AgNPs (Figure 5). The mechanism underlying these changes may be associated with the induction of the MMP signaling pathway by AgNPs. Upregulation of β-catenin probably is the endpoint of the signaling cascade through the EGFR/AKT/ERK/β-catenin axis, thus leading to an increase in the invasiveness parameter of the cells (Figure 2). ROS play a crucial role in the induction and progression of EMT. According to Jiang et al. [44], oxidative modifications of proteins possessing free thiol (-SH) groups on cysteine residues play an important role in regulating the signaling pathways. Through these redox modifications, ROS modulate the biological functions of proteins involved in ECM remodeling, for example, integrin, actin, NF-κB, HIF-1α or TGF-β, thereby regulating EMT initiation and cancer cell metastasis [45]. Zhu et al. [46] provided data on the role of heme oxygenase-1 (HMOX-1) in TGF-β-induced EMT. Hemin-induced HMOX-1 caused inhibition of migration, invasion, and ROS generation in TGB-β-induced EMT in MCF-7 cells, which once again suggests the ROS-dependent way of TGF-β action. Moreover, supplementation of the triple-negative breast cancer cell line MDA-MB-231 with resveratrol—which is a potent antioxidant—reversed the TGF-β-induced EMT [47]. Resveratrol reduced the secretion of MMP-2 and MMP-9, downregulated the expression of Smad2, Smad3 (and its phosphorylated forms), vimentin, Snail, and Slug as well as increased the expression of E-cadherin.

Snail, Slug, Twist, ZEB1, and ZEB2, responsible for repressing epithelial markers and upregulating genes associated with metastasis, are regulated by the nuclear factor-κB (NF-κB), hypoxia-inducible factor 1 (HIF-1), and transforming growth factor beta (TGF-β) signaling pathways [48]. Additionally, FoxO (forkhead box class O) transcription factor can modulate extracellular matrix (ECM) remodeling and cell mobility by promoting the expression of MMPs [49]. Furthermore, β-catenin dissociates from E-cadherin to translocate to the nucleus and bind with TCF/LEF to activate the transcription of Snail, Twist, and MMP-7 [50]. Activation of the NF-κB transcription factor induces the expression of Twist1, Snail, Slug, and ZEB1/2, all of which are involved in the disruption of cell–cell junctions [51]. Moreover, NF-κB promotes the transcription of vimentin and MMP family members. The action of NF-κB especially depends on the ROS levels—it has been reported that increased ROS levels activate NF-κB signaling pathways, thus leading to the induction of EMT, which was reversed by the addition of N-acetylcysteine- or NF-κB-specific inhibitors [52]. However, despite more evidence about the activation of NF-κB by ROS, NF-κB signaling can also be inhibited by ROS. For example, glutathionylation of p50 (a member of the NF-κB family) at Cys62 in the nucleus leads to the inhibition of the DNA-binding ability of the p50 [44]. Another transcription factor—TGF-β—plays an important role in regulating cell proliferation and adhesion. Additionally, TGF-β has a predominant role in the regulation of cell EMT through the suppression of E-cadherin expression by the activation of Snail [53]. Activated TGF-β can bind with its receptor, resulting in the phosphorylation of Smad2 and Smad3, which interact with Smad4 and trigger its translocation to the nucleus to initiate the transcription of target genes [44]. Transcriptional activation of TGF-β, mediated by the p53/SMAD/p300 complex, can be stimulated by ROS through the phosphorylation of p53 on Ser15 [54]. It is commonly known that damaged mitochondria produce excess ROS, and it has already been reported that AgNPs induce mitochondria damage [11]. Leakage of ROS from (especially damaged) mitochondria can active TGF-β signaling through the activation of TGF-β expression [55]. Secretion of TGF-β bound with latency-associated protein (LAP) is necessary for this transcription factor to react with its receptors [56]. It has been reported that LAP is sensitive to ROS; thus, it oxidizes and loses its ability to bind TGF-β, leading to the activation of TGF-β-related signaling pathways [57]. Moreover, TGF-β has been reported to promote ROS production via the disruption of oxidative phosphorylation at complex IV [58] and the regulation of antioxidant balance through the depletion of GSH, one of the major endogenous antioxidants [44]. Martin et al. [59] confirmed that AgNP exposure interferes with basement membrane and cell adhesion dynamics.

Histone deacetylation plays an important role in the development of cancer cells [60]. Inhibition of HDAC by histone deacetylase inhibitors (HDI) is often used as supportive therapy, in addition to conventional therapeutics [61]. Treatment with HDI induces MET by unblocking E-cadherin repression in cancer cells [62]. However, contradictory results were also published, in which HDI induced EMT rather than inhibiting metastasis [63]. It seems that the endpoint of HDI’s action highly depends on the type and origin of the cells as well as the type of HDI used. Vorinostat (suberanilohydroxamic acid; SAHA) is a member of the HDI family that is used to treat cutaneous T-cell lymphoma. Treatment with SAHA inhibits the EMT induced by TGF-β1 in cell lines that do not express Erα—MzChA-1 and TFK-1 [61]—via inhibition of p-SMAD2, p-SMAD3, and SMAD4 nuclear translocation induced by TGF-β1 and the partial binding inhibition of SMAD4 to E-cadherin-related transcription factors [64]. There are also reports of SAHA promoting EMT through the HDAC8/FOXA1 axis in triple-negative MDA-MB-231 and BT-549 breast cancer cells, in which SAHA upregulated the mesenchymal markers N-cadherin, vimentin, and fibronectin and downregulated E-cadherin expression [61]. Another HDI—LBH589—reversed EMT in triple-negative breast cancer cells, while no changes were noted in the ER-positive MCF-7 cells [65]. Other HDIs, such as entinostat and MS-27, have also been reported to reverse EMT in breast cancer cells; however, all of the cell lines used in these papers were triple-negative (MDA-MB-231 and Hs578T, MDA-MB-468, respectively) [66,67], although despite the aforementioned reports, the effect of HDI on cells of other origins was quite consistent. The contradictory results of HDI action are published mainly in the case of breast cancer cells. Another example of the EMT-promoting effect is a paper published by Debeb et al. [68], in which two hormone-independent cell lines (SUM159 and MDA-231) treated with valproic acid (VPA) and SAHA showed an increase in migration potential and the upregulation of epithelial markers (fibronectin, vimentin, N-cadherin and tenascin-C), whereas the E-cadherin was not detected. Additionally, the HDAC inhibition resulted in the activation of the Wnt/β-catenin signaling. The AgNPs in our results were inhibited by approximately 20% overall HDAC activity in hormone-dependent MCF-7 cells (Figure 9). We also noted an increase in β-catenin levels (Figure 5). Thus, this may suggest that AgNPss induce an effect similar to VPA in hormone-dependent breast cancer cells. It has been reported that AgNPs modulate β-catenin signaling in neural stem cells, leading to disruption of the formation of cytoskeletal inclusions [69]. Loss of E-cadherin expression results in the release of β-catenin and its translocation to the nucleus in colorectal cancer cells [70]. During EMT, the expression of β-catenin is elevated. In the present study, after 24 h of incubation, MCF-7 cells treated with 25 µg/cm^3^ AgNPs showed an increased expression of β-catenin compared to the control (*p* < 0.05; Figure 5). Moreover, the expression of E-cadherin varied significantly; however, this is not an indication that the EMT did not occur. It has been reported that cells treated with AgNPs show increased expression of the Snail protein, which has been widely described as an EMT marker [71]. Expression of the Snail protein is induced, inter alia, by the Wnt pathway, which, in turn, is associated with the expression of the β-catenin protein, thus, leading to EMT [72]. During the EMT induced by TGF-β, Snail forms a transcriptional repressor complex with SMAD3/4, targeting genes that encode junction proteins, such as E-cadherin, resulting in gene repression [73]. In the presence of Wnt signaling, β-catenin acts as a transcriptional factor through interaction with TCF/LEF, activating EMT by inducing the expression of Axin2, which stabilizes Snail [71,74]. Through the induction of Snail and β-catenin expression, the Wnt pathway provides the cells with the ability to metastasize. In 2012, Lee et al. [75] discovered a novel function of tumor progression (mediated by the Wnt pathway) that suppresses mitochondrial respiration by inhibiting cytochrome C oxidase activity and initiating the consumption of glucose, leading to a glycolytic switch.

The most probable primary mechanism for the transport of AgNPs into MCF-7 cells is clathrin-dependent endocytosis [76]. The cytotoxicity induced by AgNPs (of 50 and 100 nm in diameter) in *Mytilus galloprovincialis* is eliminated by blocking the clathrin-dependent endocytosis pathway via the addition of amantadine [77]. This could confirm the mechanism proposed by AshaRani et al. for the transport of AgNPs. Furthermore, a study by Totta et al. [78] reported that clathrin is connected with E2-signaling. The study highlighted a connection between the clathrin heavy chain (CHC) and E2-signaling and revealed that the N-terminal of the CHC directly contacts the ligand-binding domain of ERα. The findings of the present study corroborate these results.

To confirm our hypothesis that AgNPs induce EMT, the expression of primary EMT markers (Vimentin, N-Cadherin, Claudin-1, β-Catenin, ZO-1, Snail, Slug, ZEB1, E-cadherin) was analyzed via Western blot (Figure 5). In our experiments, E-cadherin, β-Catenin, Snail, and ZO-1 (with β-actin as a reference standard) were expressed by MCF-7 cells (Figure 5). MCF-7 cells cultured by Choo et al. [79] for 4 months (40 passages) were chronically exposed to a low dose of AgNPs (0.13 and 1.33 µg/cm^3^) to assess the carcinogenic potential of the nanoparticles. Chronic exposure to AgNPs resulted in a significant increase in cell migration and induced EMT. Moreover, a significant decrease in Caspase-3, which has been reported to regulate the metastasis of colon cancer cells [80], was observed, while MMP-9, which is also thought to be involved in EMT [39], was upregulated. Choo et al. suggested that long-term exposure to AgNPs could enhance cell transformation through regulation of the MAPK kinase complex [79].

As reported by Dhasarathy et al. [81], the loss of ERα in breast cancer cells is correlated with an increased incidence of metastasis. ERα is responsible for direct activation of metastasis-associated protein 3 (MTA3), which is a component of the gene repressing the histone deacetylation Mi-2/NuRD complex in breast epithelial cells [82]. One of the genes targeted by this complex is Snail [81]. Therefore, the absence of ERα (which is activated by E2) or MTA3 (activated by ERα) results in deactivation of the Mi-2/NuRD complex, leading to the aberrant expression of Snail. Activated Snail regulates EMT, leading to the repression of E-cadherin and resulting in metastasis. The mechanism underlying this process is dependent upon the activation or deactivation (in the case of no ligand) of ERα, leading to the activation of the Wnt/β-catenin pathway and/or the deactivation of the Mi-2/NuRD complex.

The present study was conducted to assess whether AgNPs could induce EMT in breast cancer cells. It is widely acknowledged that metastasis (a result of EMT) leads to the formation of new tumors, which, in turn, often results in the death of the patient. AgNPs are commonly added to cosmetics (such as antiperspirants), bandages, and fabrics, resulting in daily human contact with AgNPs. Therefore, it is important to understand the exact mechanism of action of the nanoparticles, and counteracting negative effects is an extremely important concern in modern toxicology. It is anticipated that the research presented herein will contribute to the development and understanding of the mechanisms of action of AgNPs, which are very complex and different in cells from different organs and/or species. In the present study, we wanted to check whether the addition of AgNPs will cause metastasis in hormone-dependent breast cancer cells. The estrogen-deprivation setup was meant to simulate the situation of postmenopausal women dealing with breast cancer. Our results, confronted with the current knowledge about AgNPs, suggest that AgNPs modulate a lot of different pathways in ER-positive breast cancer cells. The AgNPs showed an effect similar to HDI (Figure 9), but with the induction rather than inhibition of EMT, upregulated the expression of MTA3 and some of the EMT markers (Figure 5 and Figure 6), and caused an increase in migration capacity (Figure 2) without causing any significant drop in the cell’s survival (Figure 1). Treatment with AgNPs also induced the generation of ROS (Figure 3). Summarizing the above, we suggest that AgNPs, through the induction of ROS generation, cause a significant change in breast cancer cells. However, more data are needed to clearly state the mechanism of induction of EMT.

## 4. Materials and Methods

### 4.1. Preparation of the AgNP Solution

According to the methods outlined in our previous study [83], the AgNPs, with a nominal diameter of 20 nm, were suspended in PBS containing 1% albumin. Full characteristics, including the hydrodynamic radius of nanoparticles, of the used AgNPs have been described in the previous paper by Zuberek et al. [83].

### 4.2. Cell Culture

Cells were cultured and passaged as recommended by the American Type Culture Collection (ATCC). The MCF-7 cells were cultured in DMEM (25 mmol/dm^3^ glucose) supplemented with 10% fetal bovine serum.

Cell passage was performed twice per week after the cells reached a density within the range of 5 × 10^5^ − 6 × 10^5^/cm^2^, which corresponded to 85% cell culture confluence. The cells were passaged at a subcultivation ratio of 1:4.

### 4.3. Determination of Cell Viability Using the SRB Method

Cell viability under the influence of AgNPs was determined using the SRB fluorescent dye test based on the protocol developed by Vichai and Kirtikara [84]. For the experiments, the trypsin-released cells were transferred to 96-well flat-bottom plates (Thermo Fisher Scientific, Nunclon ™ Delta Surface, Waltham, MA, USA) at a concentration of 5 × 10^3^ cells per well in a volume of 0.1 cm^3^ of culture medium. Twenty-four hours after seeding, the AgNP solution was added to the cells in 0.1 cm^3^ of culture medium to achieve a final concentration range of 0.39–100 μg/cm^3^. After the 24 h incubation period, 0.1 cm^3^ of chilled 10% trichloroacetic acid (Sigma Aldrich, St. Louis, MO, USA) was added directly to the wells, and the plates were incubated again for 1 h at 4 °C to fix the cells to the plate. The wells were then rinsed four times with tap water and allowed to dry at room temperature. For staining, 0.1 cm^3^ per well of 0.057% SRB solution (Invitrogen, Waltham, MA, USA) in 1% acetic acid was added to the dried cells in the wells, and the plates were incubated for 30 min at room temperature. The dye solution was subsequently removed from the wells, and each well was washed four times with 1% acetic acid at a volume of 0.1 cm^3^. The plates were left to dry at room temperature. Once dried, 0.2 cm^3^/well of a 10 mmol/dm^3^ Tris solution (pH 10.5) was added, and the plates were shaken for 5 min. The fluorescence was then measured at 488 nm excitation and 585 nm emission wavelengths using an EnVision^®^ microplate reader (PerkinElmer, Waltham, MA, USA).

### 4.4. Wound Healing Test

The cells were seeded in a 6-well plate (Thermo Scientific, Nunclon ™ Delta) at a concentration of 5 × 10^5^ cells/well in a volume of 3 cm^3^ of culture medium. Once the cell cultures reached 100% confluency, a scratch was created in the cell monolayer. The monolayer was then washed three times with PBS solution, and 3 cm^3^ of cell culture medium was added. The AgNP solution was added to the wells to produce a final concentration of 25 μg/cm^3^. Afterward, photographs were taken under a Nikon (Tokyo, Japan) light microscope in the location where each scratch was made. The cells were then incubated for 24 h. After the incubation time had elapsed, photographs were taken again under a light microscope at the location of each scratch. To analyze the results, before and after images of the surface area of the scratches were compared using Nikon Ti-U Eclipse software.

### 4.5. Measurement of Free Radical and Reactive Oxygen Species Generation

Reactive oxygen species measurement was performed according to [85] and [86]. MCF-7 cells were seeded on Nunc™ 96 black flat-bottom well plates (Thermo Fisher Scientific) at a density of 5000 cells per well in 0.1 cm^3^ of full growth medium and left for 24 h for proper bottom attachment. Subsequently, 0.1 cm^3^ of AgNP solution in full medium was added into each well (to the final concentration in the wells: 50, 25, and 12,5 μg/cm^3^). Cells were then incubated for 24 h. Next, cells were washed with 0.1 cm^3^ of PBS solution, and 0.1 cm^3^ of fluorescent probe solution in PBS was added into the wells. For H_2_DCF-DA (2′,7′-dichlorodihydrofluorescein diacetate) (Thermo Fisher Scientific) and DHE (dihydroethidium) (Thermo Fisher Scientific) assays, the cells were measured every 60 s for 30 min, immediately after addition of the probe solution. For the DHR123 (dihydrorhodamine 123) (Thermo Fisher Scientific) assay, the medium containing an AgNP solution after a preincubation was removed and stored in fresh Eppendorf (Hamburg, Germany) tubes; the cells were stained with 0.1 cm^3^ of 10 μM DHR123 in PBS for 30 min in 37 °C, then washed once with PBS and covered with 0.15 cm^3^ of conditioned medium containing AgNPs and were left for 24 h incubation. Measurements of H_2_DCF-DA and the DHE signal were performed with a final concentration of the probe and filter sets Excitation/Emission (nm) as follows: 10 μM (485/535) and 5 μM (525/590). As for the DHR123 assay, the cells were measured with the 485/535 filter set. All measurements were performed with a proper dichroic mirror. Data were calculated as a curve slope value (H_2_DCF-DA and DHE assays) or endpoint value (DHR123 assay) and normalized with cell viability using the sulforhodamine B assay (described earlier). Data were collected with a Perkin Elmer Wallac EnVision 2102 multilabel reader.

### 4.6. Cell Cycle Analysis Using Flow Cytometry

The cells were seeded in a 6-well plate (Thermo Fisher Scientific, Nunclon ™ Delta) at a concentration of 4 × 10^5^ cells/well in a volume of 3 cm^3^ of culture medium. Twenty-four hours after seeding, the AgNP solution was added to the cell cultures to produce a final concentration of 25 μg/cm^3^, and the cell cultures were incubated for a further 24 h under standard conditions. The culture medium was then removed from above the cell monolayer, and 0.5 cm^3^ of 0.25% trypsin solution was added to each of the wells. When all cells had detached from the surface of the culture vessel, 1.5 cm^3^ of culture medium was added to the culture to neutralize the trypsin. The cells were then transferred to 2 cm^3^ Eppendorf tubes, and the cell suspension was centrifuged (100 rpm, 20 °C, 7 min). The supernatant was removed, and the cell pellet was resuspended by adding 0.15 cm^3^ of PBS solution. Next, 0.1 cm^3^ of the cell suspension was injected under the surface of 1 cm^3^ of a 70% aqueous ethanol solution that had been cooled to −20 °C while vortexing. The cell suspension in ethanol was centrifuged for 10 min at 3000 rpm and 4 °C. The supernatant was removed, and the pellet was resuspended in chilled PBS and washed by centrifugation for 10 min at 3000 rpm and 4 °C. The supernatant was once again removed, and the pellet was resuspended by gently pipetting in a propidium iodide solution (Invitrogen) at a concentration of 75 μmol/dm^3^, with the addition of 50 Kunitz units/cm^3^ of RNase A (Sigma Aldrich) in PBS. The cells were incubated for 30 min in the dark at 37 °C. The samples were then placed on ice, and a low flow rate cytofluorimetric measurement was taken using an LSRII instrument (BD). The data were analyzed with FlowJo software.

### 4.7. Determination of the Expression Level of Selected EMT Marker Proteins and the MTA3 Protein Using the Western Blot Method

The cells were seeded in a 6-well plate (Thermo Fisher Scientific, Nunclon™ Delta) at a concentration of 5 × 10^5^ cells/well in a volume of 3 cm^3^ of culture medium. After 24 h of incubation, the AgNP solution was added to the culture to produce a final concentration of 25 μg/cm^3^, and the cells were incubated for an additional 24 h under standard conditions. The culture medium was removed from above the cells, and the cell monolayer was rinsed with PBS solution. The cells were then lysed by adding 0.15 cm^3^/well of M-PER lysis solution (Thermo Fisher Scientific), supplemented with Halt Protease Inhibitor Cocktail protease solution (Thermo Fisher Scientific), and shaking the plates for 5 min. The cell suspension was transferred to clean Eppendorf tubes, and the samples were maintained on ice until the end of the preparation. The samples were then sonicated with an ultrasonic homogenizer and centrifuged for 5 min at 14,000 rpm and 4 °C. The supernatant was transferred to new Eppendorf tubes. To determine the protein concentration, standard curves for bovine albumin were prepared, and test samples were incubated with Pierce’s reagent (Thermo Fisher Scientific) for 5 min. Absorbance measurements were then taken using an EnVision^®^ microplate reader (PerkinElmer). Samples were prepared in a volume of 0.025 cm^3^ containing 10 µg of protein with loading buffer (Thermo Fisher Scientific) and were heat-inactivated at 95 °C for 5 min; 4–20% gradient gels (Bio-Rad, Hercules, CA, USA) were used with a Precision Plus Protein ™ Dual Color mass marker (Bio-Rad). Electrophoresis was performed in SDS-PAGE buffer (25 mmol/dm^3^ Tris, 192 mmol/dm^3^ glycine, 0.1% SDS, pH 8.3) at 130 V for approximately 1 h. After the electrophoresis was completed, the proteins were transferred from the polyacrylamide gel to the Trans-Blot^®^ Turbo™ Mini nitrocellulose membranes (Bio-Rad) using the Trans-Blot^®^ Turbo™ Transfer System (Bio-Rad). The membranes were washed three times for 5 min with TBST buffer (20 mmol/dm^3^ Tris, 150 mmol/dm^3^ NaCl, 0.1% Tween 20). The membranes were then incubated for 1 h in blocking buffer (5% powdered milk in TBST) and subsequently rinsed three times in TBST solution for 5 min. The membranes were cut into strips corresponding to individual proteins and placed in separate wells in 2 cm^3^ of blocking buffer. Corresponding antibodies were added to the membranes (see Table 1), and the wells were incubated overnight at 4 °C. The following day, a β-actin monoclonal antibody (Sigma Aldrich) was added to the membrane for 1 h. All membranes were rinsed three times in TBST solution for 5 min before the addition of 2 cm^3^ of blocking buffer. II° antibodies were added to the EMT marker proteins (Cell Signaling) and β-actin (Sigma Aldrich) and incubated for 1 h. The membranes were then washed, as outlined above, and incubated for 5 min in the imaging solution (Thermo Fisher Scientific). Images were taken using an UVITEC Cambridge Alliance HD4 Mini chemiluminescence analysis device (UVITEC, Cambridge, UK). Sample analysis was performed using UVITEC Alliance software.

The same methodology was used for the measurement of MTA3 protein levels in the cell lysates.

### 4.8. Mitochondrial Membrane Potential Measurement

Analysis of mitochondrial membrane potential was performed with Mitotracker™ Red CMXRos (Thermo Fisher Scientific), which is a non-ratiometric probe with a signal corresponding to a membrane potential value [87]. After the incubation with the AgNP, as described in Section 4.5, the medium was removed from the wells. Then, the cells were covered with 0.1 cm^3^ of 200 nM Mitotracker™ Red CMXRos solution in PBS and incubated for 30 min at 37 °C. After staining, the cells were washed once with PBS and covered with 0.1 cm^3^ of the buffer solution. Data were collected with a Perkin Elmer Wallac EnVision 2102 multilabel reader (Perkin Elmer) with filter set Ex/Em: 570/590 and then normalized with sulphorhodamine B assay measurement results (described earlier). All values were reduced by the value collected from the stained empty well, which corresponds to the unspecific binding of a probe to a surface of the plate’s well. Conditions of staining were tested in a preliminary study using fluorescent-inverted microscopy.

### 4.9. Measurement of the Intracellular Calcium Flux in MCF-7 Cells Treated with AgNPs

Measurement of intracellular calcium flux was performed using a Fura-2, AM ratiometric fluorescent probe (Invitrogen™, Thermo Fisher Scientific). MCF-7 cells were seeded on a Nunc™ 96 black flat bottom well plate (Thermo Fisher Scientific) at the density of 5000 cells per well in 0.1 cm^3^ of full growth medium and left for 24 h for proper bottom attachment. Next, cells were stained with 5 μM probe solution in PBS (without Ca^2+^ and Mg^2+^) for 30 min at 37 °C. After that, the cells were washed with PBS solution. Every well was covered with 0.1 cm^3^ of the buffer and measured before incubation to check the basal signal. The experiment started with the addition of 0.1 cm^3^ of appropriate PBS buffer into control wells and AgNP solution into the treatment wells (final concentration 25 μg/cm^3^). The fluorescence signal was measured for 2 h every 15 min. The fluorescence of the probe was induced by 340 and 380 nm excitation light and measured at 510 nm emission wavelength. The data are presented as a 340/380 nm ratio and were analyzed by GraphPad Prism 8.

### 4.10. Determination of the Histone Deacetylase (HDAC) Activity

Measurement of HDAC activity was performed using a HDAC Activity Fluorometric Kit provided by Cayman Chemical (Ann Arbor, MI, USA). The experiment was divided into two parts: extraction of the nuclei from MCF-7 cells and measurement of HDAC activity.

Extraction of the nuclei: the cells were seeded in 75 cm^3^ culture flasks to provide approximately 1 × 10^7^ cells for lysis. When the culture achieved 75–80% confluence, an AgNP solution in 1x PBS was added to the final concentration of 25 μg/cm^3^, and the cells were incubated for 24 h in standard conditions. Next, the culture medium from above the cells was discarded, and the cells were washed once with 1x PBS solution and trypsinized. After that, the cell suspensions were centrifuged at 100× *g* for 10 min, the supernatant was discarded, and the cells were resuspended in 1 cm^3^ of cold lysis buffer. Next, the samples were vortexed for 10 s and kept on ice for 15 min. The cells were then centrifuged through 4 cm^3^ of cold sucrose cushion (following the instruction) at 800× *g* for 10 min at 4 °C. The centrifuge force was lowered from 1300 to 800× *g* due to the low quality of the samples caused by higher centrifugation force. The supernatant was discarded, and the nuclei pellet was resuspended in 1 cm^3^ of cold 10 mM Tris-HCl, pH 7.5 (containing 10 mM NaCl). A sample of the nuclei extract was visualized with Hoechst 33,342 under a Nikon Ti-U Eclipse fluorescence microscope to ensure that the samples contain only the nuclei. The samples were then centrifuged at 800× *g* for 10 min at 4 °C, and the supernatant was discarded. The precipitates were suspended in 0.15 cm^3^ of extraction buffer. The samples were sonicated for 30 s and left on ice for 30 min. After that, the samples were centrifuged at 10,000× *g* for 10 min at 4 °C, and the supernatant was stored at −80 °C until use.

Measurement of HDAC activity: The 96-well plate was prepared according to the manufacturer’s protocol. The fluorescence signal was measured using an excitation wavelength of 340 nm and an emission wavelength of 450 nm.

## Figures and Tables

**Table 1:** Antibodies used for protein detection via the Western blot method; r—rabbit protein, m—mouse protein.

Antigen	Antibody Clone 1° (Manufacturer)	Antibody Isotype	Antibody 2°
β-catenin	D10A8 (Cell Signaling)	Rabbit IgG_1_	Goat anti-rIgG_1_ conjugate with HRP
E-cadherin	24E10 (Cell Signaling)	Rabbit IgG_1_	Goat anti-rIgG_1_ conjugate with HRP
Snail	C15D3 (Cell Signaling)	Rabbit IgG_1_	Goat anti-rIgG_1_ conjugate with HRP
ZO-1	D7D12 (Cell Signaling)	Rabbit IgG_1_	Goat anti-rIgG_1_ conjugate with HRP
ZEB1	D80D3 (Cell Signaling)	Rabbit IgG_1_	Goat anti-rIgG_1_ conjugate with HRP
Claudin-1	D5H1D (Cell Signaling)	Rabbit IgG_1_	Goat anti-rIgG_1_ conjugate with HRP
β-actin	AC-74 (Sigma Aldrich)	Mouse IgG_1_	Rabbit anti-mIgG1 conjugate with HRP
MTA3	428C2a (Santa Cruz Biotechnology)	Mouse IgG_1_	Rabbit anti-mIgG1 conjugate with HRP

## Data Availability

The data presented in this study is available on request from the corresponding author.
